# A Novel Validated UHPLC Method for the Estimation of Rosuvastatin and Its Complete Impurity Profile in Tablet Formulations

**DOI:** 10.3390/molecules28010431

**Published:** 2023-01-03

**Authors:** Francesca Romana Mammone, Leo Zanitti, Michela Puxeddu, Giuseppe La Regina, Romano Silvestri, Anna Borioni, Roberto Cirilli

**Affiliations:** 1 Centre for the Control and Evaluation of Medicines, Chemical Medicines Unit, Istituto Superiore di Sanità, Viale Regina Elena 299, 00161 Rome, Italy; 2 Department of Drug Chemistry and Technologies, Sapienza University of Rome, P.le Aldo Moro 5, 00185 Rome, Italy

**Keywords:** rosuvastatin calcium salt, impurities, European pharmacopoeia, UHPLC, method validation, reversed-phase, tablets

## Abstract

A key step in the development of medicinal products is the research and validation of selective and sensitive analytical methods for the control of impurities from synthesis and degradation. As most impurities are similar in structure to the drug substance, the achievement of chemo-selective conditions is usually challenging. Herein, a direct and highly selective ultra-high-performance liquid chromatographic method for determining the assay and related substances content in medicinal products containing rosuvastatin calcium salt (RSV) is presented. RSV is used to treat high cholesterol levels and prevent heart attacks and strokes. The most engaging feature of this method was the baseline separation of all organic related substances listed in the European Pharmacopoeia (EP) monograph for the RSV tablets, achieved for the first time in less than 15 min using the Acquity BEH C18 (100 mm × 2.1 mm, 1.7 μm) column under reversed-phase isocratic conditions. The mobile phase adopted for the chemo-selective analysis does not contain buffers but instead contains trifluoroacetic as an acid additive. The chromatographic method was validated according to the guidelines of the International Conference on Harmonization (ICH) and proved to be linear, precise and accurate for determining the content of RSV and related chiral substances in tablet formulations.

## 1. Introduction

Most pharmaceutical products are produced by applying a total synthesis approach or by modifying a naturally occurring product. In both cases, a wide range of organic substances can be produced in the manufacturing process and during the subsequent storage and may persist as unavoidable impurities in the finished product. Although their levels are usually very low (i.e., concentration < 0.5%), impurities are an unwanted burden on metabolism and may have toxic effects, including genotoxicity. Accordingly, the control of impurities in active pharmaceutical ingredients (APIs) represents a topic of interest for pharmaceutical companies, regulatory authorities and patients [1]. Organic impurities cannot be completely eliminated, but the efforts of the pharmaceutical industry should be focused on markedly reducing their concentration to levels that constitute a minor risk to patients. In this context, the characterization, detection, and quantification of impurities related to the API play a vital role in ensuring the safety and efficacy of the drug [2]. To investigate the quality of APIs in drugs, regulatory agencies and pharmaceutical industries usually apply chromatographic methods reported in the monographs of the reference pharmacopeias. However, due to the fast continuous progress in technology, the analytical protocols developed years ago are now far outdated in terms of costs, analysis times and efficiency. Typically, a complete analysis of an API in a medicinal product involves at least two separation methods, one for the API content and the other for related substances. This usually requires a minimum of two different columns and two separate chromatographic runs in addition to equilibration times for the two columns. A convenient and sophisticated approach to simplify the analytical procedure is to use a single highly selective and efficient chromatographic column packed with sub-2 µm (UHPLC) stationary phase particles as the column packing material [3]. The employment of smaller particle chromatographic packing materials allows improvements in terms of efficiency, resolution, sensitivity and analysis time [4], as well as being more environmentally friendly.

The control of organic molecules as impurities in APIs is especially important in the case of blockbuster medications such as statins, which are used by a large population group for a long time [5,6].

**Figure 1:**
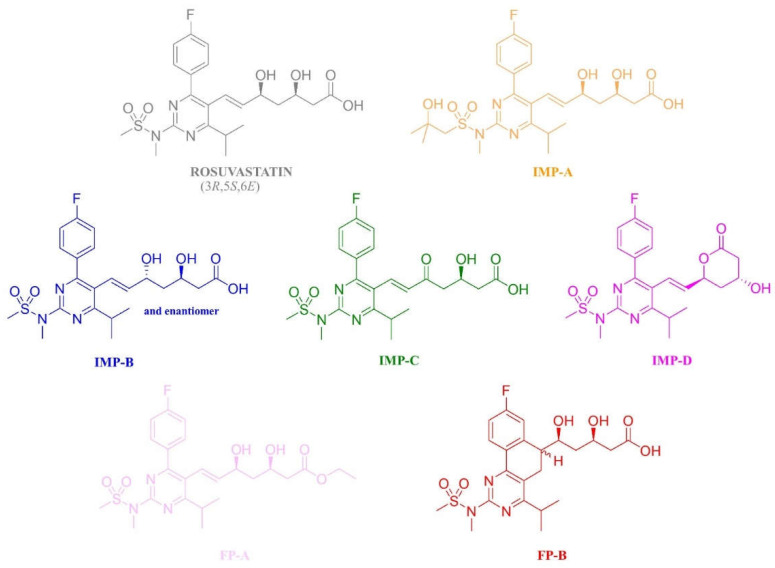
Chemical structures and abbreviations of rosuvastatin and its potential organic impurities listed in the EP monograph for rosuvastatin tablets. IMP-A: impurity A; IMP-B: impurity B; IMP-C: impurity C; IMP-D: impurity D; FP-A: impurity FP-A; FP-1: first eluted diastereomer of the impurity FP-B; FP-2: second eluted diastereomer of the impurity FP-B.

Statins are used for the treatment of hyperlipidemia because of their capability to inhibit the enzyme 3-hydroxy-3-methylglutarylcoenzyme A (HMG-CoA) reductase, which catalyzes the conversion of HMG-CoA to mevalonate; this is the rate-limiting step of cholesterol biosynthesis in the liver. One of the many statins registered by regulatory agencies worldwide [7] is the fully synthetic chiral statin rosuvastatin (RSV) (Figure 1), chemically named as (3*R*,5*S*,6*E*)-7-[4-(4-fluorophenyl)-6-(1-methylethyl)-2-[methyl(methylsulfonyl)amino]-5-pyrimidinyl]-3,5-dihydroxy-6-heptenoicacid, calcium salt (2:1) [8]. RSV is available in 5, 10, 20 and 40 mg tablet dosages. The structures of the potential RSV-related substances, either its degradants or synthetic by-products, are reported in the rosuvastatin tablets monograph in the European Pharmacopoeia (EP) [9] and are shown in Figure 1. The current EP monograph HPLC method for the determination of the API content uses a 150 mm × 3.0 mm column packed with 3.5 µm C18-bonded silica particles under a reversed-phase elution gradient mode. 

Usually, specificity is not an issue in the validation of analytical methods for the assay of finished products, as the impurity content is negligible (usually less than 0.5%) compared to the API content (acceptance criteria is in the range 95–105%). In the case of RSV, since the limit for the total impurity content in tablets is 2.5%, the coelution of organic impurities with the API could significantly alter the results of the assay. For this reason, quantitative analysis of the RSV content in finished products should be carried out according to a selective chromatographic protocol.

In the EP monograph, impurity quantification in RSV tablets is carried out by using the same column described for the assay but with different reversed-phase gradient elution. Two main problems are encountered with this method. Firstly, the run time analysis is fairly high (i.e., 46 min). Secondly, the peak of IMP-B falls on the tail of the peak of RSV. Therefore, the integration of these two adjacent non-equimolar peaks results in some inaccuracy.

Different chromatographic methods have been reported in the literature for the quantitative analysis of RSV impurities [10,11,12,13,14,15,16]. Typically, they were developed for identifying and separating degradation products formed under various forced conditions (acid and alkaline hydrolysis as well as oxidative and photolytic stress) using reversed-phase C18 packing materials.

However, to the best of our knowledge, a suitable protocol capable of determining all the related substances listed in the EP monograph in a single chromatographic run in RSV-based formulations has not been reported yet. 

In light of all the arguments presented, the challenge in the present work is in providing a simple, rapid, accurate and sensitive single-run UHPLC method to simultaneously separate and quantify the impurities of RSV and determine the content of API in the tablet formulations with full selectivity. The effect of the mobile phase composition and temperature has been evaluated to optimize chemo-separation properties of the selected chromatographic system.

## 2. Results and Discussion

### 2.1. UHPLC Separation under Reversed-Phase Conditions

ACQUITY UPLC™ BEH is a commercially available family of columns based on a bridged ethylsiloxane/silica hybrid (BEH) structure. The Acquity BEH C18 column utilizes highly efficient 1.7 µm C18 bonded stationary phase particles and it is used to improve the speed, sensitivity and resolution of chromatographic separations. Reddy et al. [13] and Trivedi et al. [14] developed a UHPLC method based on the Acquity BEH C18 (100 mm × 2.1 mm, 1.7 μm) column for the determination of some impurities of RSV in pharmaceutical preparations. 

Reddy et al. [13] report a reversed-phase UHPLC method for the simultaneous separation of RSV, its related impurities IMP-B, IMP-C and IMP-D, as well as impurities resulting from forced degradation of API. The mobile phase was a 50:50 (*v*/*v*) mixture of methanol and trifluoroacetic acid (TFA) 0.1%. 

The UHPLC method proposed by Trivedi et al. [14] proved to be successful in separating a multi-component chemical mixture containing six unknown degradation products formed under acidic and oxidative stress conditions, three known impurities (IMP-B, IMP-C and IMP-D) and RSV within 10 min. Gradient elution using a mobile phase of TFA 0.1% and methanol was employed. Both reversed-phase UHPLC methods were validated according to the International Conference on Harmonization (ICH) guidelines and applied for the quantitative determination of impurities in tablet formulations.

**Figure 2:**
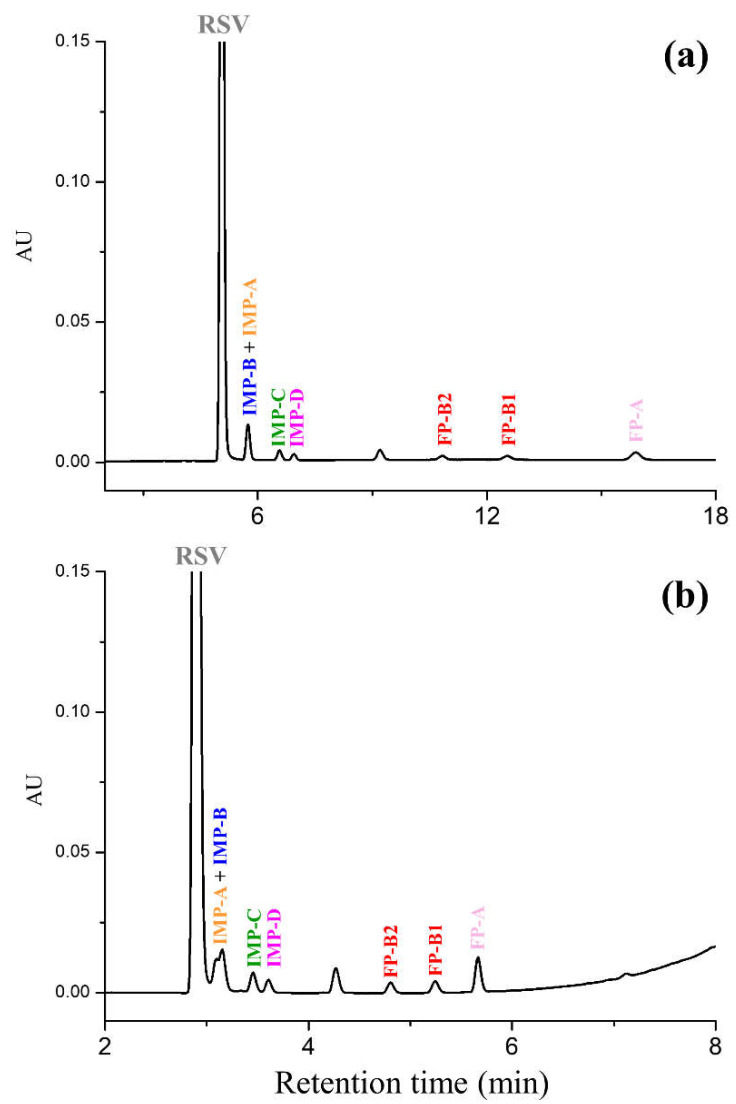
Comparison between UHPLC chromatograms of RSV spiked with impurities on the Acquity BEH C18 (100 mm × 2.1 mm, 1.7 μm) column using (**a**) isocratic and (**b**) gradient elution. Chromatographic conditions: mobile phase, (**a**) MeOH-TFA 0.1% 50:50 (*v*/*v*), (**b**) solvent A (0.1% TFA (*v*/*v*) in water) and solvent B (MeOH) [14]; temperature, 40 °C; flow rate, 0.3 mL/min; detection, UV at 240 nm. RSV: rosuvastatin; IMP-A: impurity A; IMP-B: impurity B; IMP-C: impurity C; IMP-D: impurity D; FP-A: impurity FP-A; FP-1: first eluted diastereomer of the impurity FP-B; FP-2: second eluted diastereomer of the impurity FP-B; MeOH: methanol; TFA: trifluoroacetic acid.

Figure 2 shows the typical chromatograms resulting from the UHPLC analysis of RSV spiked with all known impurities listed in EP monograph carried out utilizing the two published reversed-phase methods based on the BEH C18 column.

As can be seen, in gradient elution mode, the impurities IMP-A and IMP-B coelute and the merged peak falls on the RSV peak tail, whereas the same organic substances are only poorly resolved (Rs < 1) in the isocratic run. All other impurities were well separated with resolution values above 1.5.

To search for more specific conditions, we decided to introduce some subtle and accurate changes to the chromatographic parameters governing the separation carried out by the protocol proposed by Reddy et al. In particular:

(i) The TFA percentage was reduced from 0.1 to 0.025% with the purpose of preventing the potential on-column degradation of RSV.
(ii) The methanol percentage was reduced from 50 to 45%, this change gave rise to a partial resolution of the critical pair IMP-B/IMP-A (Rs = 1.24) and an increase in the analysis time to 36 min.
(iii) The column temperature and flow rate were increased from 40 to 55 °C and 0.3 to 0.5 mL/min, respectively. Under these conditions, all impurities were baseline separated from each other and eluted before 15 min.

**Figure 3:**
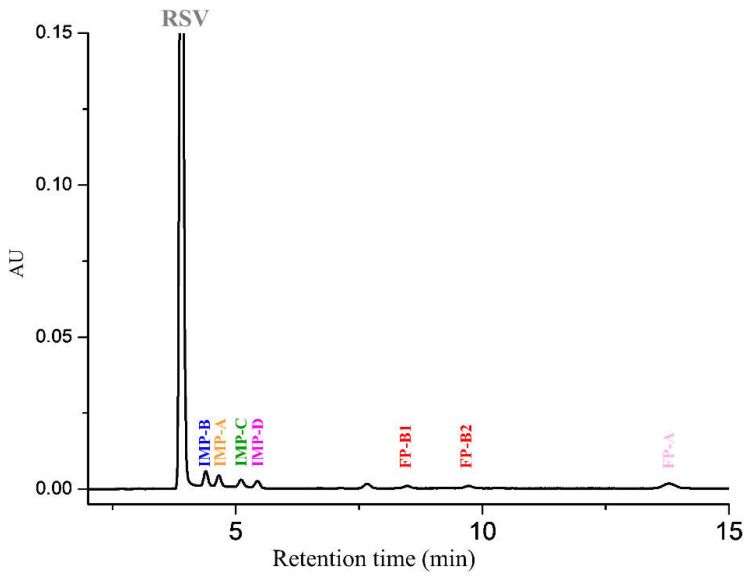
Optimized UHPLC chromatogram of RSV spiked with impurities. Chromatographic conditions: column, Acquity BEH C18 (100 mm × 2.1 mm, 1.7 μm); mobile phase, MeOH-TFA 0.025% 45:55 (*v*/*v*); temperature, 55 °C; flow rate, 0.5 mL/min; detection, UV at 240 nm. RSV: rosuvastatin; IMP-A: impurity A; IMP-B: impurity B; IMP-C: impurity C; IMP-D: impurity D; FP-A: impurity FP-A; FP-1: first eluted diastereomer of the impurity FP-B; FP-2: second eluted diastereomer of the impurity FP-B; MeOH: methanol; TFA: trifluoroacetic acid.

The outcomes of such changes are shown in Figure 3. 

The analysis of the chromatographic data summarized in Table 1 revealed that the peak belonging to diastereoisomer IMP-B was baseline resolved from the main RSV peak (Rs = 3.23) and the IMP-A peak (Rs = 1.60). Furthermore, it is worth noting that the two epimers originating from photolytic degradation of RSV are well separated, with a resolution of 3.73 (Table 1). Overlapping chromatograms of placebo, diluent and RSV at limit of quantitation (LOQ) (Figure S1 of Supplementary Materials) demonstrated that no peak interference disturbed the analysis.

The novelty and advantages of the chromatographic protocol herein presented can be summarized as follows:

(i) Absence of buffer in the mobile phase; as a consequence, the risk of formation of precipitates in the column due to the poor solubility of salts in the organic modifier is overcome;
(ii) Rapid elution of all the substances, both active substances and impurities, present in the multicomponent sample;
(iii) High selectivity with excellent chromatographic resolution between adjacent peaks.

After establishing a fundamental aspect of impurity analysis, namely the specificity, it remained to validate the single-run UHPLC method according to ICH guidelines and to demonstrate that its characteristics (system suitability, precision, linearity accuracy, robustness and LOQ) were suitable for the quantitative determination of impurities in RSV tablet formulation.

### 2.2. Method Validation

#### 2.2.1. System Suitability

The RSV for system suitability standard (RSV-SS) was employed to establish the resolution between RSV and the critical impurities IMP-A, IMP-B and IMP-C. 

As shown in Table 1, the resolution values between the critical pairs RSV/IMP-B, IMP-B/IMP-A and IMP-A/IMP-C were higher than the required limit of 1.5.

Standard assay (0.4 mg/mL) and related substance (0.02 mg/mL) solutions were analyzed six times to evaluate the repeatability of the method. As highlighted in Table 2: (i) the percentage of relative standard deviation (RSD) of peak area and retention times for both standard solutions were below 0.4% (the acceptance criterion is that the RSD% is less than 2.0%); (ii) peak tailing was not more than 1.10 (the acceptance criterion is that the peak tailing is less than 1.5); and (iii) theoretical plates were about 11,000 (the acceptance criterion is that the theoretical plates are more than 10,000). 

#### 2.2.2. Linearity

The linearity of the UHPLC protocol was assessed by analyzing RSV solutions with concentrations ranging from 1000 µg/mL to 0.59 µg/mL (100% and 0.06% of concentration used for impurities determination) for RSV free acid. The linearity was calculated by linear regression analysis by plotting the peak area against the concentration (mg/mL). The obtained calibration curve equation is 4216859.31x − 1493.51 and the correlation coefficient R^2^ = 0.9999 (acceptable value: R^2^ not lower than 0.9990). The obtained calibration curves for impurities were: y = 3052128.93x + 4.42 (R^2^ = 0.9999) for IMP-A free acid (y is the peak area and x concentration (mg/mL)); y = 3686295.70x + 2361.55 (R^2^ = 1.0000) for IMP-B free acid; y = 1954417.57x + 474.86.55 (R^2^ = 1.0000) for IMP-C free acid; y = 4015579.67x − 3740.83 (R^2^ = 0.9993) for IMP-D free acid; y = 3713429.25x − 3786.79 (R^2^ = 1.0000) for FP-A; and y = 2764448.85x + 1283.45 (R^2^ = 1.0000) for FP-B1 free acid. 

The relative response factors reported in Table 1 were calculated by the ratio of slopes of linearity plots for the impurity and API.

#### 2.2.3. Limits of Detection and Quantitation

The ratio values between the RSV peak signal and the level of background noise (S/N) of 3 and 10 were used as a measure for the determination of the limit of detection (LOD) and limit of quantitation (LOQ) values, respectively. The LOQ value corresponds to 0.59 µg/mL (S/N = 13), whereas the LOD was equal to 0.18 µg/mL. The LOQ/LOD values for impurities ranged from 0.62/0.19 to 0.72/0.22 µg/mL.

#### 2.2.4. Accuracy

Table 3 shows the results of the recovery expressed in terms of RSD%. The percentage recovery obtained for RSV at five concentration levels (i.e., <1%) indicates that the proposed UHPLC method is accurate (acceptable value: 98–102%).

#### 2.2.5. Precision and Repeatability

The precision of the chromatographic protocol was established by repeatability (intra-day) and intermediate precision (inter-day) tests. Regarding RSV, the method exhibited RSD% lower than 0.4% for retention times and peak areas. The obtained values met the proposed acceptance criteria (acceptable value: RSD% not higher than 2.0%). Regarding impurities, the method gave the following RSD% for retention times and peak areas: between 0.2% and 0.4% for repeatability and between 0.5 and 1.3% for intermediate precision. The repeatability and intermediate precision data meet the proposed acceptance criteria (RSD% not higher than 5.0%). 

### 2.3. Determination of RSV and Impurity Contents in Commercial Tablets

**Figure 4:**
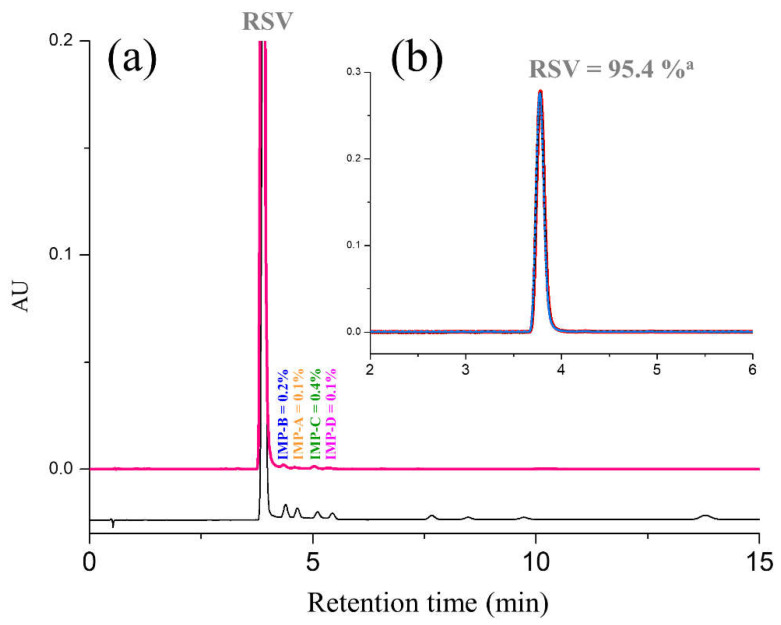
(**a**) Comparison of chromatograms obtained from the analysis of a test solution for impurities (pink line) and an RSV resolution solution (grey line); (**b**) typical chromatograms obtained from the analysis of test solution for assays (*n* =3). Chromatographic conditions: column, Acquity BEH C18 (100 mm × 2.1 mm, 1.7 μm); mobile phase, MeOH-TFA 0.025% 45:55 (*v*/*v*); temperature, 55 °C; flow rate, 0.5 mL/min; detection, UV at 240 nm. ^a^ Mean value of triplicate analysis. RSV: rosuvastatin; IMP-A: impurity A; IMP-B: impurity B; IMP-C: impurity C; IMP-D: impurity D; MeOH: methanol; TFA: trifluoroacetic acid.

The optimized UHPLC method was applied to analyze marketed tablets containing 10 mg of RSV expressed as a free acid. Figure 4a shows a comparison between the chromatograms illustrating the analysis of impurities and the multi-component RSV resolution solution. 

To assess the assay of RSV free acid in the tablets, the three test solutions used for determining the impurities in tablets were diluted 4:10 and analyzed in the same chromatographic conditions (Figure 4b). The data obtained from UHPLC analysis, which are reported in Figure 4, revealed an RSV content of 95.4%, 0.4 % of IMP-C and 0.1% of IMP-A and IMP-D (in triplicate, RSD% < 0.3%).

## 3. Materials and Methods

### 3.1. Chemical and Reagents

TFA (99.5%) and HPLC-grade water and methanol were supplied by Aldrich (Milan, Italy) and filtered through a 0.22 μ filter. RSV and RSV-SS standards were purchased from the European Directorate for the Quality of Medicines and Healthcare (EDQM) (France). The impurities A (potency 96.7%, 3*R*,5*S*, HCl salt, IMP-A), B (potency 97.5%, 3*R*,5*R*, HCl salt, IMP-B), C (potency 100% (3*R*), IMP-C), D (potency 89.3% (3*R*,5*S*), HCl salt, IMP-D), FP-B (as a mixture of epimers, potency 44% for the first eluted epimer and potency 48% for the second eluted epimer) (Figure 1) and a placebo of Crestor 10 mg film-coated tablets were kindly provided by AstraZeneca (Macclesfield, UK). Crestor 10 mg film-coated tablets were purchased from a local drugstore. 

Chromatographic analyses were carried out on the Acquity UPLC BEH C18 column (100 mm × 2.1 mm, 1.7µm, Waters, Milford, MA, USA). 

### 3.2. Instruments

The UHPLC system used for separations under reversed-phase conditions consisted of a Waters Acquity UPLC system (Waters, Milford, MA, USA), equipped with a quaternary solvent manager, sample manager, an Acquity photodiode array detector with a 1.0 μL internal volume flow cell and a column heater. Data acquisition was performed using Empower software.

^1^H NMR and ^13^C NMR spectra were recorded on a Bruker AvanceNeo 600 MHz (Bruker Biospin, Ettlingen, Germany) using MeOH-d4 as solvent. Chemical shifts were defined as δ values in ppm (parts per million) referenced to the residual solvent signal (^1^H 3.33; ^13^C 49.5). ^1^H NMR spectra were described as follows: δ chemical shift/ppm (multiplicity, number of protons, J-coupling). ^13^C NMR spectra were described as follows: δ chemical, shift/ppm, carbon. Multiplicity is defined as follows: s, singlet; d, doublet; t, triplet; q, quartet; m, multiplet; dd, doublet of doublets. The value of coupling, J, is in hertz (Hz).

Melting points (mp) were determined on a Stuart Scientific SMP1 (Bibby Scientific Ltd., Staffordshire, UK) apparatus and were not corrected. Infrared (IR) spectra were recorded on a PerkinElmer Spectrum 100 FT-IR (Norwalk, CT, USA) spectrophotometer equipped with a universal attenuated total reflectance accessory and IR data were acquired and processed by PerkinElmer Spectrum 10.03.00.0069 software. Band positions and absorption ranges are given in cm^−1^. 

Mass spectra were recorded on a Thermo Scientific (Bremen, Germany) LTQ mass spectrometer with an electrospray ionization (ESI) source. Compound purity was checked by high-pressure liquid chromatography (HPLC). The purity of tested compounds was found to be >95%. The HPLC system used (Thermo Fisher Scientific Inc. Dionex UltiMate 3000) consisted of an SR-3000 solvent rack, an LPG-3400SD quaternary analytical pump, a TCC-3000SD column compartment, a DAD-3000 diode array detector and an analytical manual injection valve with a 20 μL loop. Samples were dissolved in acetonitrile (1 mg/mL). To check the purity of FP-A, an HPLC analysis was performed by using a Thermo Fisher Scientific Inc. Acclaim 120 C18 column (5 μm, 4.6 mm × 250 mm) at 25 ± 1 °C with an appropriate solvent gradient (acetonitrile/water), a flow rate of 1.0 mL/min and a signal detector at 206, 230, 254 and 365 nm. Chromatographic data were acquired and processed by Thermo Fisher Scientific Inc. Chromeleon 6.80 SR15 Build 4656 software.

### 3.3. Synthesis of the Impurity FP-A

All reagents and solvents used in the synthesis of the impurity, FP-A, were handled according to the material safety data sheet of the supplier and were used as purchased without further purification. Organic solutions were dried over anhydrous sodium sulfate. Evaporation of solvents was carried out on a Büchi Rotavapor R-300 equipped with a Büchi V-850 (Flawil, Switzerland) vacuum controller, a Büchi V-300 vacuum pump and a recirculating chiller, F-305. Column chromatography was performed on columns packed with silica gel from Macherey-Nagel (70–230 mesh). Silica gel thin layer chromatography (TLC) cards from Macherey-Nagel (silica gel precoated aluminum cards with fluorescent indicator visualizable at 254 nm) were used for TLC. Developed plates were visualized with a Spectroline ENF 260C/FE UV apparatus. 

Rosuvastatin calcium (0.500 g, 1.0 mmol) was dissolved in *N,N*-dimethylformamide (2.5 mL), then iodoethane (0.156 g, 1.0 mmol) was added and the mixture was heated at 45 °C for 5 h. The reaction mixture was cooled, diluted with water and extracted with ethyl acetate. The organic layer was washed with brine, dried and filtered. Evaporation of the solvent gave a residue that was purified by column chromatography (silica gel, c-hexane:acetone = 65:45) to give FP-A (0.240 g, 47%), mp 103–107 °C. 

The structure of impurity FP-A was confirmed by ^1^H, ^13^C NMR, IR and MS data. ^1^H NMR (CD_3_OD, 600 MHz): 1.27 (t, 3H), 1.31 (d, 6H), 1.53–1.57 (m, 1H), 1.67–1.72 (m, 1H), 2.49–2.41 (m, 2H), 3.49–3.54 (m, 1H), 3.53 (s, 3H), 3.56 (m, 3H), 3.99–4.03 (m, 1H), 4.14–4.19 (m, 2H), 4.35–4.38 (q, 2H), 5.57 (dd, 1H, J_16.1,Hz,_ J_6.3 Hz_), 6.66 (dd, 1H, J_16.1,Hz,_ J_1.1 Hz_), 7.19 (t, 2H), 7.74 (dd, 2H); ^13^C NMR (CD_3_OD, 600 MHz): 14.94 (CH_3_), 22.51 (CH_3_), 33.71 (CH), 34.25 (CH_3_), 42.77 (CH_3_), 43.85 (CH_2_), 45.11 (CH_2_), 62.03 (CH_2_), 67.60 (CH), 71.52 (CH), 116.5 (d, CH, J_C-F 21.9 Hz_), 123.59 (Cq), 124.90 (CH), 133.92 (CH, d J_C-F 8.7 Hz_), 136.62 (Cq, d, J_C-F 2.9 Hz_), 142.07 (CH), 159.28 (Cq), 165.19 (Cq, d, J_C-F 247.9 Hz_), 165.17 (Cq), 173.66 (Cq), 176.89 (Cq). IR v 1721 and 3424 cm^−1^. MS (ESI): 510.25 (MH+). C_24_H_32_FN_3_O_6_S (509.20).

### 3.4. Method Validation

#### 3.4.1. HPLC Operating Conditions

Analytical chromatographic separations were performed on an Acquity UPLC BEH C18 column (100 mm × 2.1 mm, 1.7 µm, Waters, Milford, CT, USA) with a mobile phase consisting of methanol–TFA (0.025%) 55:45 (*v*/*v*) at a flow rate of 0.5 mL/min and maintaining the column temperature at 55 °C. The injection volume was 0.9 μL and the sampler temperature and the detection wavelength were 20 °C and 240 nm, respectively. 

#### 3.4.2. Specificity

The selectivity of the analytical method was evaluated by the analysis of the system suitability solution containing RSV and its related substances. A system suitability solution was prepared by dissolving 2.0 mg of RSV-SS standard in 20 mL volumetric flasks with diluent (methanol:water 50:50). The specificity was established by analyzing the resolution solution and single solutions of each impurity. The resolution solution was prepared as follows: (i) 2.0 mg of impurities A, B, C, D or FP-A were weighed in a 10 mL volumetric flask and dissolved with diluent up to volume (c = 0.2 mg/mL); (ii) 2.0 mg of the mixture of two epimers FP-B were weighed in a 5 mL volumetric flask and dissolved with diluent up to volume (c = 0.2 mg/mL for each epimer); (iii) an accurately weighed amount of RSV, which was equivalent to about 20 mg of RSV free acid, was transferred into a 50 mL volumetric flask and dissolved in 40 mL of diluent. The solution was spiked with 1.0 mL of impurity solutions and then diluted up to volume with diluent (concentration of RSV = 0.4 mg/mL, concentration of impurities = 0.008 mg/mL (1%)).

#### 3.4.3. Sample Solutions to Determine the Content of Rosuvastatin and Its Impurities

To determine the content of RSV and its impurities in Crestor 10 mg film-coated tablets, 20 tablets containing 10.0 mg of RSV as API were accurately weighed and the average mass was calculated. The tablets were made into a fine powder and the powder equivalent to 100 mg of RSV was transferred into a 100 mL volumetric flask to which the diluent was added up to volume. The dispersion was stirred for 20 min and an aliquot was filtered through a 0.2 µm nylon filter. This solution was employed as a sample solution for determining the RSV impurities (c = 1.0 mg/mL). Four mL of this solution was diluted to 10 mL with methanol and filtered. The resulting solution was used for determining the RSV assay (c = 0.4 mg/mL).

#### 3.4.4. Standard Stock Solutions

Standard solutions of RSV were prepared as follows: (i) an accurately weighed amount of RSV, which was equivalent to about 20 mg of RSV free acid, was transferred into a 50 mL volumetric flask; (ii) 25 mL of methanol was added and the resulting solution was maintained in an ultrasonic bath for 5 min and then diluted up to volume with water; (iii) an aliquot of this solution was filtered through a 0.2 µm nylon filter. This solution was employed as an assay standard (c = 0.4 mg/mL, corresponding to 10 % of the sample solution). Five milliliters of this assay standard solution was diluted up to 100 mL with diluent (a mixture methanol–water 50:50, *v*/*v*) and used as the standard to quantify the RSV impurities (c = 0.02 mg/mL, corresponding to 0.5% of sample solution).

#### 3.4.5. Linearity

The linearity evaluation was performed with the standard solutions of RSV free acid at concentrations ranging from 1000 µg/mL to 0.59 µg/mL. Three injections of each solution were made under the chromatographic conditions described above, using an injection volume of 0.9 µL. The concentrations of solutions were plotted against the corresponding peak areas response of RSV free acid and the linear regression equations were computed.

Linearity tests for impurities were performed with a standard solution containing IMP-A, IMP-B, IMP-C, IMP-D, FP-A and FP-B at concentrations ranging from 210–180 to 21–18 µg/mL.

#### 3.4.6. Limits of Detection and Quantitation

LOD and LOQ are the concentration of the analyte that would yield a S/N ratio of 3 and 10, respectively, following the EP guidelines. The LOD and LOQ of RSV and related impurities were determined by injecting a series of diluted solutions.

#### 3.4.7. Precision

Method precision was assessed by measuring the repeatability (intra-day precision) and intermediate precision (inter-day precision) of peak areas and retention times for RSV. The intra-day variability was carried out by the same analyst over one day, while inter-day precision was performed by another independent analyst and by using a different HPLC apparatus over three days. The precision was determined by measuring the repeatability of retention time and peak areas in replicate injections (*n* = 6). Precision was reported as the percentage of relative standard deviation (RSD%).

## 4. Conclusions

A simple, reproducible and linear isocratic reversed-phase UHPLC method based on the Acquity UPLC BEH C18 column (100 mm × 2.1 mm, 1.7µm) was developed for the separation and accurate quantification of all impurities of RSV listed in the pertinent EP monograph. It presents more selective properties compared to other published procedures which use the same chromatographic column, due to its ability to separate RSV and impurities without overlapping peaks in a single chromatographic run. The single-run protocol has been successfully validated and applied for the quantitative determination of RSV and organic related substances contained in commercial 10 mg film-coated tablets. The improved selectivity and sensitivity as well as its time- and solvent-saving characteristics distinguish the presented UHPLC method from that reported in the EP monograph and it could routinely be adopted in a quality control department to determine the stability and assay of RSV in commercial samples.

## Figures and Tables

**Table 1:** Relative retention times (*RRT*) of RSV and its impurities, resolution factors (*Rs*) (resolution between two adjacent peaks) and relative response factors (*RRF*). Chromatographic conditions: column, Acquity BEH C18 (100 mm × 2.1 mm, 1.7 μm); mobile phase, MeOH-TFA 0.025% 45:55 (*v*/*v*); temperature, 55 °C; flow rate, 0.5 mL/min; detection, UV at 240 nm. RSV: rosuvastatin; IMP-A: impurity A; IMP-B: impurity B; IMP-C: impurity C; IMP-D: impurity D; FP-A: impurity FP-A; FP-1: first eluted diastereomer of the impurity FP-B; FP-2: second eluted diastereomer of the impurity FP-B; MeOH: methanol; TFA: trifluoroacetic acid.

*Impurity*	*RRT*	*Rs*	*RRF*
RSV	1.00		
IMP-B	1.13	3.23	0.87
IMP-A	1.19	1.60	0.72
IMP-C	1.31	2.56	0.46
IMP-D	1.39	1.76	0.95
FP-B1	2.17	9.59	0.66
FP-B2	2.49	3.73	0.66
FP-A	3.54	9.61	0.88

**Table 2:** System suitability results (peak area, retention time, peak tailing and theoretical plates) at two different concentrations of RSV (top: 0.40 mg/mL; bottom: 0.02 mg/mL). Chromatographic conditions: column, Acquity BEH C18 (100 mm × 2.1 mm, 1.7 μm); mobile phase, MeOH-TFA 0.025% 45:55 (*v*/*v*); temperature, 55 °C; flow rate, 0.5 mL/min; detection, UV at 240 nm. RSV: rosuvastatin; MeOH: methanol; TFA: trifluoroacetic acid; RSD: relative standard deviation.

*Injection*	*Peak Area*	*Retention Time*	*Peak Tailing*	*Theoretical Plats*
1	1,689,234	3.954	1.09	11,407
2	1,694,889	3.945	1.09	11,326
3	1,689,344	3.963	1.09	11,289
4	1,693,483	3.945	1.08	11,397
5	1,689,940	3.93	1.09	11,333
6	1,686,928	3.935	1.09	11,280
RSD(%)	0.18	0.31		
1	79,897	3.94	1.07	11,386
2	79,195	3.94	1.06	11,669
3	79,755	3.94	1.08	11,356
4	79,189	3.92	1.07	11,354
5	79,708	3.92	1.06	11,548
6	79,460	3.93	1.07	11,586
RSD (%)	0.38	0.26		

**Table 3:** Accuracy results. Chromatographic conditions: column, Acquity BEH C18 (100 mm × 2.1 mm, 1.7 μm); mobile phase, MeOH-TFA 0.025% 45:55 (*v*/*v*); temperature, 55 °C; flow rate, 0.5 mL/min; detection, UV at 240 nm. ^a^ Triplicate determination at each level. MeOH: methanol; TFA: trifluoroacetic acid; RSD: relative standard deviation; LOQ: limit of quantitation.

*Amount Added*	*%Recovery*	*RSD% ^a^*
0.06% (LOQ)	100.92	0.93
0.5%	100.45	0.34
80	100.81	0.51
100	100.77	0.62
120	99.25	0.68

## Data Availability

Data sharing not applicable.

## Supplementary Materials

The following supporting information can be downloaded at: https://www.mdpi.com/article/10.3390/molecules28010431/s1, Figure S1: Typical chromatograms pertinent to placebo, RSV at LOQ value and diluent.
