# Supplementary material for: A Novel Validated UHPLC Method for the Estimation of Rosuvastatin and Its Complete Impurity Profile in Tablet Formulations
Source: Molecules. 2023 Jan 3;28(1):431. doi: 10.3390/molecules28010431 (PMC9824232; doi:10.3390/molecules28010431)

Supplementary Materials

A Novel Validated UHPLC Method for the Estimation of Rosuvastatin and Its Complete Impurity Profile in Tablet Formulations

Francesca Romana Mammone [1], Leo Zanitti [1], Michela Puxeddu [2], Giuseppe La Regina [2], Romano Silvestri [2], Anna Borioni [1] and Roberto Cirilli [1,*]

[1] Centre for the Control and Evaluation of Medicines, Chemical Medicines Unit, Istituto Superiore di Sanità, Viale Regina Elena 299, 00161 Rome, Italy
[2] Department of Drug Chemistry and Technologies, Sapienza University of Rome, P.le Aldo Moro 5, 00185 Rome, Italy
* Correspondence: roberto.cirilli@iss.it

Figure S1. Typical chromatograms pertinent to placebo, RSV at LOQ value and diluent. Chromatographic conditions: column, Acquity BEH C18 (100 mm × 2.1 mm, 1.7 µm); mobile phase, MeOH-TFA 0.025% 45:55 (v/v); temperature, 55 °C; flow rate, 0.5 mL/min; detection, UV at 240 nm.

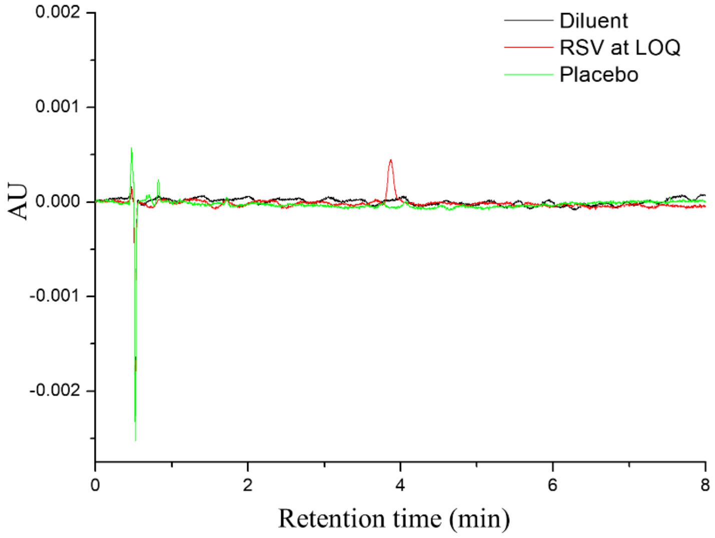

Supplement: Supplementary file 1 [file molecules-28-00431-s001.zip › molecules-2104545-supplementary.pdf]
